# Toll-like receptor 2 orchestrates a tumor suppressor response in non-small cell lung cancer

**DOI:** 10.1016/j.celrep.2022.111596

**Published:** 2022-11-08

**Authors:** Fraser R. Millar, Adam Pennycuick, Morwenna Muir, Andrea Quintanilla, Priya Hari, Elisabeth Freyer, Philippe Gautier, Alison Meynert, Graeme Grimes, Carla Salomo Coll, Sofia Zdral, Stella Victorelli, Vitor H. Teixeira, John Connelly, João F. Passos, Marian A. Ros, William A.H. Wallace, Margaret C. Frame, Andrew H. Sims, Luke Boulter, Sam M. Janes, Simon Wilkinson, Juan Carlos Acosta

**Affiliations:** 1 Cancer Research UK Scotland Centre, Institute of Genetics and Cancer, University of Edinburgh, Edinburgh EH4 2XR, UK; 2 Lungs for Living Research Centre, UCL Respiratory, University College London, London WC1E 6JF, UK; 3 Instituto de Biomedicina y Biotecnologia de Cantabria, IBBTEC (CSIC, Universidad de Cantabria), C/Albert Einstein 22, 39011 Santander, Spain; 4 MRC Human Genetics Unit, Institute of Genetics and Cancer, University of Edinburgh, Edinburgh EH4 2XR, UK; 5 Department of Physiology and Biomedical Engineering, Mayo Clinic, Rochester, MN 55905, USA; 6 Robert and Arlene Kogod Center on Aging, Mayo Clinic, Rochester, MN 55905, USA; 7 Department of Pathology, NHS Lothian, Edinburgh EH16 4SA, UK; 8 Lead contact

## Abstract

Targeting early-stage lung cancer is vital to improve survival. However, the mechanisms and components of the early tumor suppressor response in lung cancer are not well understood. In this report, we study the role of Toll-like receptor 2 (TLR2), a regulator of oncogene-induced senescence, which is a key tumor suppressor response in premalignancy. Using human lung cancer samples and genetically engineered mouse models, we show that TLR2 is active early in lung tumorigenesis, where it correlates with improved survival and clinical regression. Mechanistically, TLR2 impairs early lung cancer progression via activation of cell intrinsic cell cycle arrest pathways and the proinflammatory senescence-associated secretory phenotype (SASP). The SASP regulates non-cell autonomous anti-tumor responses, such as immune surveillance of premalignant cells, and we observe impaired myeloid cell recruitment to lung tumors after *Tlr2* loss. Last, we show that administration of a TLR2 agonist reduces lung tumor growth, highlighting TLR2 as a possible therapeutic target.

## In brief

Millar et al. show that TLR2 is expressed in early lung cancer, where it is associated with improved survival and clinical regression. Using mouse models, they show that Tlr2 regulates growth arrest pathways and anti-tumoral immune surveillance. Targeting Tlr2 activity with an agonist significantly reduces early lung tumor growth.

## Graphical abstract

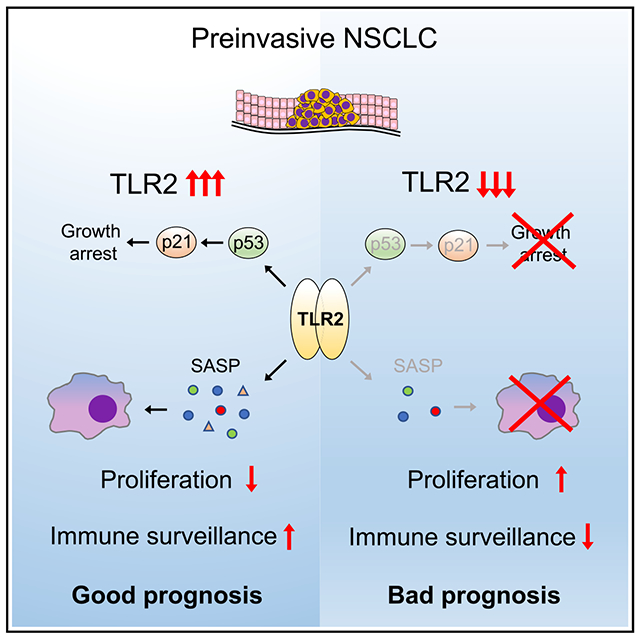

## INTRODUCTION

Lung cancer is the most lethal cancer type worldwide (Siegel et al., 2015). Non-small cell lung cancer (NSCLC) accounts for the overwhelming majority of cases with lung adenocarcinoma (LUAD) and lung squamous carcinoma (LUSC), the predominant NSCLC subtypes. Early-stage NSCLC is associated with a 5-year survival rate of over 50% (Goldstraw et al., 2016). However, the majority of patients present with advanced-stage disease, which has a 5-year survival rate of just 6% (Goldstraw et al., 2016). Recent advances with immunotherapy and targeted therapies against identifiable driver oncogenes has improved outcomes in a small subset of patients with NSCLC (Camidge et al., 2012; Gandhi et al., 2018; Mok et al., 2009). However, because of the low frequency of targetable mutations and the inevitable emergence of resistance and disease relapse, overall outcomes are still very poor. To improve lung cancer survival, it is clear that we must understand and target early-stage disease. Despite this, the overall understanding of the early tumor suppressor responses in lung cancer is poor.

Oncogene-induced senescence (OIS) is a stress response that is initiated after activation of oncogenes and is a key tumor suppressor mechanism *in vivo* (Collado and Serrano, 2010). Markers of OIS are widespread in preinvasive lung tumors in mice but are lost during progression to invasive malignancy (Collado et al., 2005). Although this suggests a tumor suppressor role for OIS in this context, the precise role of OIS in human lung cancer is unclear and requires active investigation. Along with robust cell cycle arrest mediated by p53-p21 and p16-Rb tumor suppressor pathways (Chicas et al., 2010), OIS is characterized by significant metabolic upregulation and expression of a variety of secreted factors collectively called the senescence-associated secretory phenotype (SASP) (Acosta et al., 2008; Coppe et al., 2008). Although many stressors that induce the SASP also induce the growth arrest (for example, DNA damage), the SASP can be induced independent of tumor suppressor pathways (Coppe et al., 2008, 2010; Freund et al., 2011). The SASP has wide-ranging functions, including potent pro- and anti-tumorigenic effects. Chronic inflammation driven by an overactive SASP has been shown to promote tumor growth and invasion (Krtolica et al., 2001; Liu and Hornsby, 2007; Parrinello et al., 2005), and senescence and the SASP have been recognized as a hallmark of cancer (Hanahan, 2022). However, the SASP can impair tumor progression in other contexts. It has been shown to reinforce senescence-associated growth arrest in an autocrine manner (Acosta et al., 2008), induce senescence in neighboring cells (paracrine senescence) (Acosta et al., 2013), and orchestrate immune cell-mediated clearance of senescence cells (senescence surveillance) (Kang et al., 2011; Xue et al., 2007). Thus, the effects of the SASP on tumor growth are varied and context dependent. The role of the SASP in cancer has been well demonstrated using animals models, but, to our knowledge, there are no clear data defining the role of the SASP in human cancer. The innate immune receptor Toll-like receptor 2 (TLR2) is a key regulator of OIS and the SASP (Hari et al., 2019). Canonical TLR2 activation occurs after binding to microbial lipoproteins, resulting in heterodimerization with other cell membrane TLR2 binding partners (TLR1, TLR6, or the less well-characterized TLR10). This prompts an intracellular signaling cascade that culminates in nuclear factor κB (NF-κB) activation (Oliveira-Nascimento et al., 2012). We have demonstrated previously that TLR2 is upregulated in OIS and is activated via binding to acute-phase serum amyloid A (A-SAA), a key component of the SASP and acute-phase response. This activation, in turn, regulates expression of a variety of pro-inflammatory SASP factors (Hari et al., 2019). Although we have shown that TLR2 regulates a pro-inflammatory SASP in OIS, it is not yet known whether this has a role in tumor suppression. TLR2 loss has been shown to impair immune- and senescence-related tumor suppressor effects in carcinogen-induced models of liver cancer (Lin et al., 2013), but, to date, the role of TLR2 in lung tumor progression and human disease in particular has not been investigated. Here, using human NSCLC samples and genetically engineered mouse models (GEMMs) of lung cancer, we demonstrate that TLR2 coordinates induction of cell-autonomous and non-cell-autonomous tumor suppressor responses that together impair NSCLC progression. Understanding this process identifies TLR2 as a therapeutic target for early-stage lung cancer.

## RESULTS

### *TLR2* expression correlates with improved survival and clinical regression in human lung cancer

**Figure 1.**
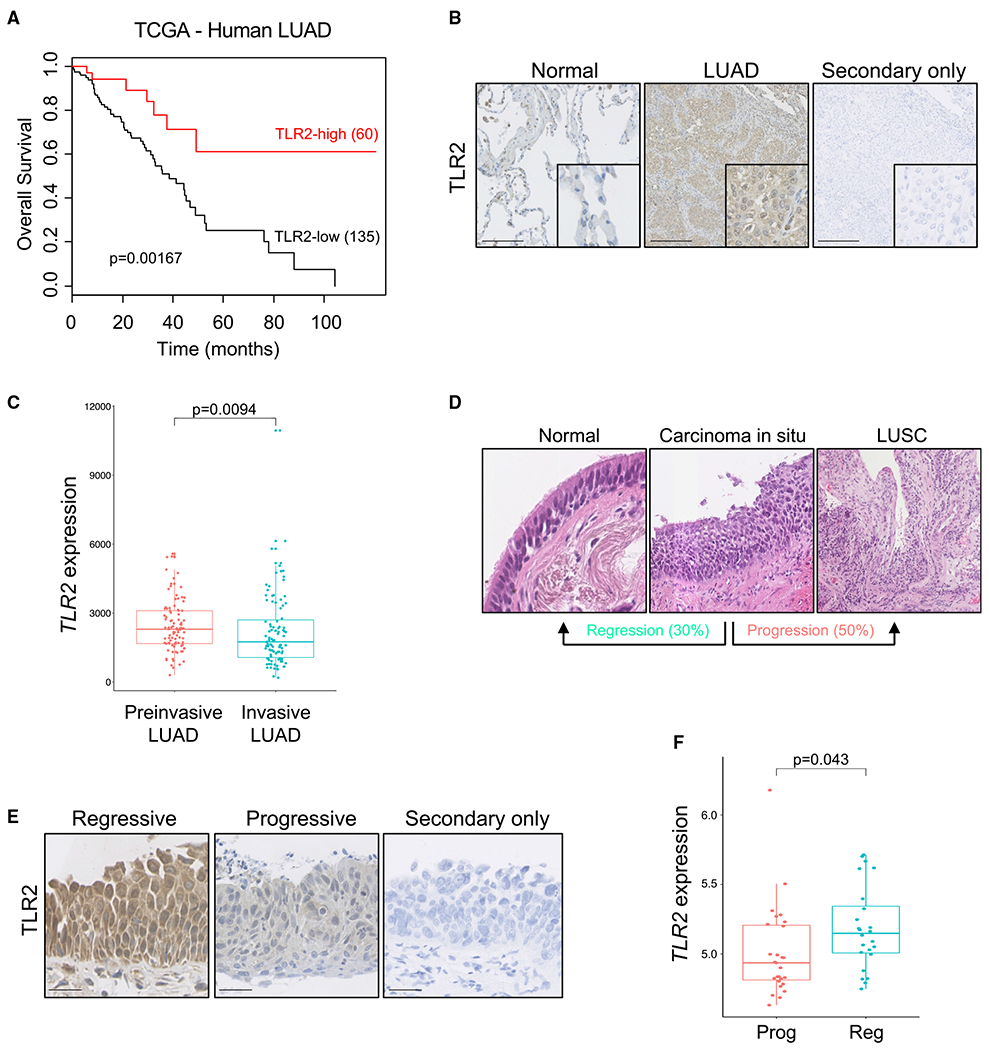
TLR2 expression correlates with improved survival and clinical regression in human lung cancer (A) Kaplan-Meier survival analysis of patients with LUAD based on high versus low *TLR2* expression from the Cancer Genome Atlas (TCGA). Statistical analysis was performed using log rank (Mantel-Cox) test. (B) Representative IHC staining for TLR2 in paired normal tissue and LUAD samples, with secondary only control. Scale bars, 250 μm. (C) TLR2 gene expression was compared between preinvasive LUAD lesions (AIS and MIA) and invasive LUAD lesions. Data presented as median +/− interquartile range (IQR). Statistical analysis was performed using the Mann-Whitney test. (D) Hematoxylin and eosin (H&E) images of preinvasive LUSC lesions, demonstrating that not all progress to invasive cancer; up to 30% regress to normal epithelium. (E) Representative IHC staining for TLR2 in preinvasive LUSC lesions that progressed to cancer or regressed to normal epithelium, with secondary only control. Scale bars, 25 μm. (F) *TLR2* gene expression was compared between preinvasive LUSC lesions of equal grade that subsequently progressed to cancer (progressive [Prog]) or regressed to normal epithelium (regressive [Reg]). Data presented as median +/− IQR. Statistical analysis was performed using a linear mixed effects model to account for multiple samples from the same patient.

We analyzed *TLR2* expression in human LUAD samples from The Cancer Genome Atlas (TCGA) (Cancer Genome Atlas Research, 2014 and found that high *TLR2* expression significantly correlated with improved survival (Figure 1A). This correlation in LUAD was also observed in genes encoding TLRs that form heterodimers with TLR2 upon activation (TLR1, TLR6, and TLR10) but not with the other plasma membrane TLRs (such as TLR4) (Figures S1A–S1C), indicating a specific role of the TLR2 sensing networks in LUAD. We then sought to cross-validate this finding in a separate tissue bioresource and simultaneously determine in which cellular compartment TLR2 is expressed. To do this, we performed immunohistochemistry (IHC) staining for TLR2 on surgically resected early-stage human LUAD specimens and found that TLR2 expression is significantly increased in LUAD tumor epithelium in comparison with adjacent normal epithelium (Figures 1B and S1D). We then wanted to explore in which stage of tumorigenesis *TLR2* expression is activated. We therefore analyzed an RNA sequencing dataset from a cohort of preinvasive (adenocarcinoma *in situ* [AIS] and minimally invasive adenocarcinoma [MIA]) and invasive LUAD samples (Chen et al., 2019). The gene expression profiles of AIS and MIA lesions were similar (Figure S1F); hence, these lesion types were grouped together for further analysis. *TLR2* gene expression was significantly increased in preinvasive LUAD tumors compared with invasive LUAD tumors (Figure 1C), suggesting that *TLR2* is induced early in lung tumorigenesis. These LUAD lesions are surgical resection specimens; therefore, we cannot glean any prognostic insight based on their *TLR2* expression profiles. To answer this question requires longitudinal sampling and clinical follow-up of individual lesions, which is not possible for surgically resected LUAD tumors. Due to their proximal location making them accessible to bronchoscopic sampling, human preinvasive LUSC lesions have undergone extensive longitudinal molecular characterization in comparison with distally located preinvasive LUAD lesions. Longitudinal follow-up of preinvasive LUSC lesions using autofluorescence bronchoscopy has shown that not all preinvasive lesions progress to invasive malignancy. In fact, only half progress to cancer, and up to 30% regress to normal epithelium (Figure 1D; Jeremy George et al., 2007). We therefore analyzed biopsy samples and transcriptomics data from a unique cohort of preinvasive LUSC samples that have been carefully clinically phenotyped, detailing subsequent progression to cancer or regression to normal epithelium (Teixeira et al., 2019). We analyzed *TLR2* expression in these samples and found that *TLR2* mRNA expression significantly correlated with subsequent clinical regression (Figures 1E and 1F), and this trend was reflected at the protein level (n = 3–4, p = 0.0546; Figure S1E). The gene expression profiles of these samples represent lesion epithelium only because stroma was removed by laser capture microdissection prior to RNA extraction. Whole-genome sequencing of these preinvasive LUSC lesions revealed that loss of heterozygosity (LOH) for *TLR2* was a common event. The likelihood of LOH being more common in progressive versus regressive lesions was analyzed using Fisher’s exact test and revealed that *TLR2* LOH is more likely in progressive lesions (p = 0.07; Figures S1G and S1H), suggesting that LOH may contribute to the reduced *TLR2* expression observed in progressive lesions. However, this result did not reach statistical significance. This effect was specific to TLR2 and its binding partners because non-TLR2 associated plasma membrane TLRs (TLR4/5) showed no association with lesion progression (Figures S1G and S1H). Although LOH may partially underlie the altered *TLR2* expression in preinvasive LUSC, such genomic changes are not evident in preinvasive LUAD (Figure S1I). We then sought to determine whether epigenetic regulation of the *TLR2* locus affects its expression in lung tumors. To do this, we analyzed the methylation patterns of the *TLR2* locus in LUAD and LUSC tumor samples from the TCGA (Cancer Genome Atlas Research, 2012, 2014) and compared them with adjacent normal lung tissue. Again, this showed no difference between normal lung and tumor (LUAD and LUSC) (Figures S1J and S1K). Despite this, we see clear differences in *TLR2* mRNA expression in early lung tumors, suggesting that *TLR2* expression is regulated at the transcriptional level. Indeed, we have shown previously *in vitro* that *TLR2* expression in the context of OIS is regulated at the transcriptional level (Hari et al., 2019).

These data demonstrate that *TLR2* is widely expressed in human NSCLC epithelium (LUAD and LUSC) and correlates with improved survival and clinical regression, respectively. It must be noted that these are distinct pathological entities with differing genetic drivers; therefore, findings in LUSC samples are not directly transferable to LUAD. Nonetheless, these data strongly suggest a potential tumor suppressor function of *TLR2* in NSCLC and suggest a common role of TLR2 in NSCLC subtypes.

### *Tlr2* impairs early tumor development in murine models of lung cancer

**Figure 2.**
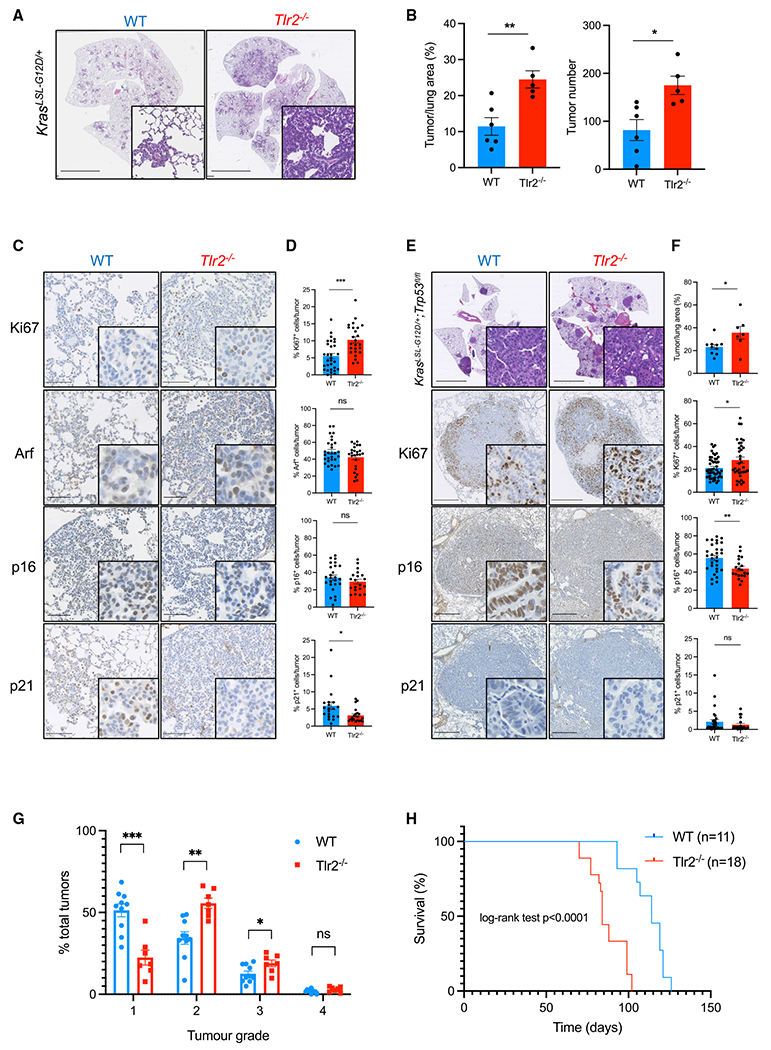
*Tlr2* has a tumor suppressor function in murine models of lung cancer (A) Representative H&E images from *Kras*^*LSL-G12D*/+^ mice on a wild-type (WT) or *Tlr2*-null (*Tlr2*^−/−^) background 12 weeks after intranasal inoculation with AdenoCre. Scale bars, 5 mm. (B) Quantification of tumor number and tumor burden (tumor area/total lung area × 100) from mice described in (A). n = 5–6 mice per group. Data presented as mean +/− standard error of the mean (SEM). Statistical analysis was performed using Student’s t test. *p < 0.05, **p < 0.01. (C and D) Representative IHC staining of lung tumors from WT or *Tlr2*^−/−^ mice for Ki67, Arf, p16, and p21 (C) with corresponding quantification (D). n = 5–6 mice per group (five tumors per mouse analyzed). Data presented as mean +/− SEM. Statistical analysis was performed using Student’s t test. ns, non-significant; *p < 0.05, ***p < 0.001. Scale bars, 100 μm. (E and F) Representative H&E and IHC staining for Ki67, p16, and p21 of lung tumors from *Kras*^*LSL-G12D*/+^;*Trp53*^*fl/fl*^ (KP) mice on a WT or *Tlr2*^−/−^ background (E) with corresponding quantification (F). n = 7–10 mice per group (five tumors per mouse analyzed). Data presented as mean +/− SEM. Statistical analysis was performed using Student’s t test. *p < 0.05, **p < 0.01. Scale bars, 5 mm for H&E images and 500 μm for IHC images. (G) Histological grading (1–4) of tumors from WT or *Tlr2*^−/−^ KP mice 12 weeks after intranasal inoculation with AdenoCre. n = 7–10 mice per group. Data presented as mean +/− SEM. Statistical analysis was performed using Student’s t test. *p < 0.05, **p < 0.01, ***p < 0.001. (H) Kaplan-Meier curve showing survival analysis of WT or *Tlr2*^−/−^ KP mice after inoculation with AdenoCre. Statistical analysis was performed using log rank (Mantel-Cox) test.

Based on our data described above, we hypothesized that TLR2 has a tumor suppressor role in early-stage lung cancer. To investigate this, we used a well-described GEMM of preinvasive lung cancer driven by Cre recombinase-mediated activation of oncogenic *Kras*^*G12D*^ (Kras^LSL–G12D/+^) (DuPage et al., 2009). After intranasal administration of a Cre recombinase-expressing adenovirus (AdenoCre), lung epithelium-specific activation of *Kras*^*G12D*^ occurs, and tumor formation is initiated. Kras^LSL–G12D/+^ mice on a *Tlr2* wild-type (WT) or *Tlr2*-null (*Tlr2*^−/−^) background were inoculated with AdenoCre, and lung tissue was harvested 12 weeks later for histological analysis. *Tlr2* knockout (KO) in the lung was confirmed by mRNA and protein expression analysis (Figures S2A and S2B). Kras^LSL–G12D/+^ mice on a *Tlr2*^−/−^ background (*Kras*^LSL-G12D/+^;*Tlr2*^−/−^) developed more tumors and had a significantly increased tumor burden compared with controls (*Kras*^*LSL-G12D*/+^;*Tlr2*^+/+^) (Figures 2A and 2B). *Tlr2*^−/−^ lung tumors exhibited a higher proliferation index, as assessed by IHC staining for Ki67 (Figures 2C and 2D). To investigate whether the increased proliferation caused by *Tlr2* loss was due to impaired OIS, we analyzed markers of key senescence-associated events, such as p53 induction and *Cdkn2a* locus activation (Barradas et al., 2009; Chicas et al., 2010). The cell cycle inhibitors p21 (a product of *Cdknla* and indicator of p53 transcriptional activity), Arf (the alternate reading frame of the *Cdkn2a* locus), and p16 (a product of *Cdkn2a*) were analyzed in lung tumors by IHC. *Tlr2*^−/−^ tumors exhibited significantly reduced expression of p21 in comparison with controls, but there was no significant difference in *Cdkn2a* expression (Figures 2C and 2D), suggesting that p53-p21 signaling is central to *Tlr2*-mediated proliferative arrest in this context. We then wanted to assess whether the overall tumor suppressor activities of *Tlr2* were dependent on p53-p21 signaling. To do this, we used a GEMM where mice not only possess the conditional *Kras*^*G12D*^ allele but also have *loxP* sites flanking the *Trp53* allele (*Kras*^*LSL-G12D*/+^;*Trp53*^*fl/fl*^, so-called “KP” mice; DuPage et al., 2009). Therefore, administration of AdenoCre in KP mice not only activates oncogenic *Kras*^*G12D*^ signaling but also deletes *Trp53* at the lung epithelium. Tumor burden was also significantly increased in KP mice on a *Tlr2*^−/−^ background (*Kras*^*LSL-G12D*/+^; *Trp53*^*fl/fl*^;*Tlr2*^−/−^) (Figures 2E and 2F). Furthermore, *Tlr2* loss increased proliferation in KP lung tumors (Figures 2E and 2F) and resulted in tumors of a significantly more advanced histological grade (Figure 2G). Survival of *Tlr2*^−/−^ KP mice after tumor initiation was also significantly reduced compared with controls (Figure 2H). We also analyzed p21 and p16 expression in these KP tumors. p21 expression was essentially absent in KP tumors regardless of *Tlr2* status (Figures 2E and 2F), suggesting that the increased p21 expression we observed in *Tlr2*-proficient *Kras*^*LSL-G12D*/+^ lung tumors (Figure 2C) is p53 dependent. p16 expression, however, was significantly reduced in *Tlr2*^−/−^ KP tumors, suggesting that, in the absence of *Trp53*, Tlr2 inhibits tumor growth by activating p16 signaling. These data support the hypothesis that *Tlr2* loss accelerates lung tumor progression. This is in part due to alteration in p21 expression, but the persisting tumor suppressor effect of *Tlr2* after *Trp53* loss suggests that this effect of *Tlr2* is not entirely dependent on cell-autonomous p53-p21 signaling.

### *Tlr2* expression controls the inflammatory response during NSCLC initiation

**Figure 3.**
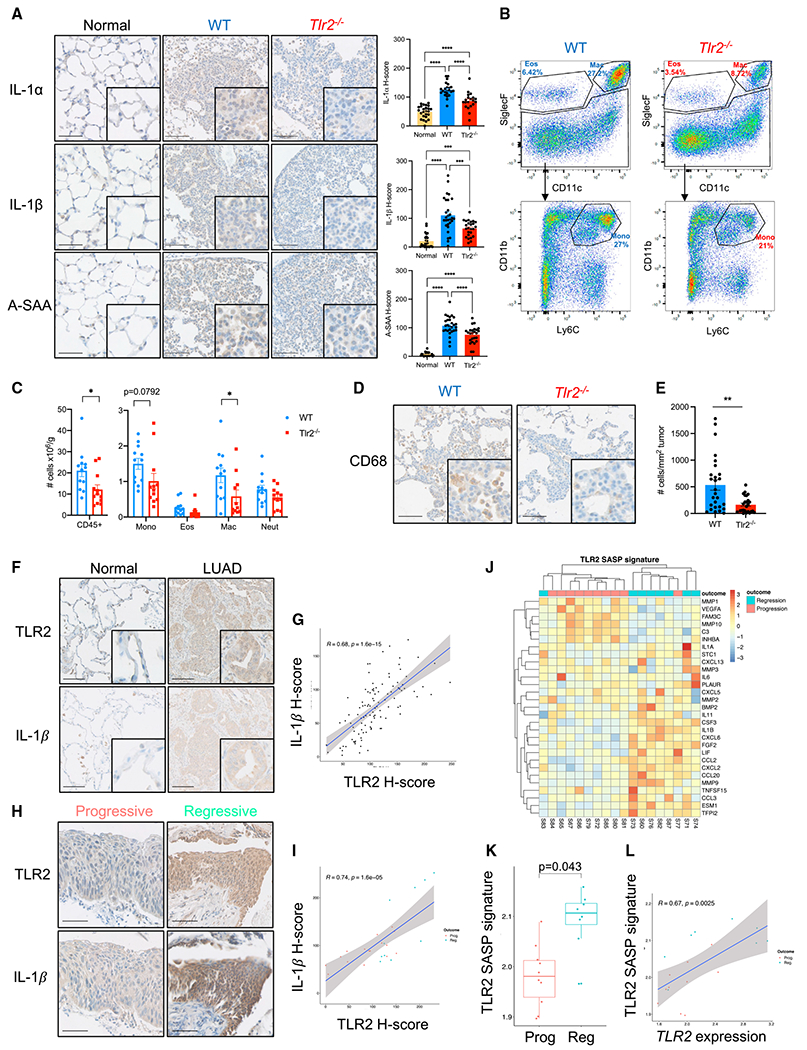
*Tlr2* loss impairs expression of the senescence-associated secretory phenotype (SASP) in lung tumors (A) Representative IHC staining of normal alveolar tissue (normal) and lung tumors from *Kras*^*LSL-G12D*/+^ mice on a WT or *Tlr2*^−/−^ background for the SASP factors interleukin-1-alpha (IL-1*α*), interleukin-1-beta (IL-1*β*), and serum amyloid A (SAA), with corresponding quantification. n = 5–6 mice per group (five tumors/areas per mouse analyzed). Data presented as mean +/− SEM. Statistical analysis was performed using a one-way ANOVA with post hoc Tukey tests for multiple comparisons. ***p < 0.001, ****p < 0.0001. Scale bars, 100 μm. (B) Representative flow cytometry analysis plots of myeloid populations from whole-lung single-cell suspensions from tumor-bearing WT or *Tlr2*^−/−^ mice. Percentage denotes the percentage of the parent population. (C) Corresponding quantification of total immune cells (CD45^+^) and myeloid cells from WT (blue) and *Tlr2*^−/−^ (red) mice. Mono, monocyte; Eos, eosinophils; Mac, macrophage; Neut, neutrophils. n = 12 mice per group. Data presented as mean +/− SEM. Statistical analysis was performed using Student’s t test. *p < 0.05. (D and E) Representative IHC staining for the monocyte/macrophage marker CD68 in lung tumors from WT or *Tlr2*^−/−^ mice (D) with corresponding quantification (E). Data presented as mean +/− SEM. Statistical analysis was performed using Student’s t test. **p < 0.01. Scale bars, 100 μm. (F) Representative IHC staining for TLR2 and IL1*β* in consecutive sections of LUAD and paired normal tissue. Scale bars, 100 μm. (G) Scatterplot with Pearson correlation analysis of IHC H-score analysis performed on serial sections for TLR2 and IL-1*β*. (H) Representative IHC staining for TLR2 and IL-1*β* in preinvasive LUSC lesions that progressed to cancer or regressed to normal epithelium. Scale bar, 25 μm. (I) Scatterplot with Pearson correlation analysis from IHC H-score analysis performed on serial sections for TLR2 and IL-1*β* on preinvasive LUSC lesions that progressed to cancer or regressed to normal epithelium. (J) Heatmap demonstrating TLR2-SASP gene expression with clear clustering of progressive and regressive lesions. (K) The TLR2-SASP signature was compared between lesions of equal grade that subsequently progressed to cancer or regressed to normal epithelium. Data presented as median +/− IQR. Statistical analysis was performed using a linear mixed-effects model to account for samples from the same patient. (L) Scatterplot with Pearson correlation analysis comparing gene expression of TLR2 and the TLR2-SASP signature in Prog and Reg lesions.

A key non-cell-autonomous facet of OIS is expression of the SASP (Acosta et al., 2008; Coppe et al., 2008; Kuilman et al., 2008). The role of the SASP is complex and context dependent, and the SASP has been shown to have pro and anti-tumorigenic effects (Coppe et al., 2010; Faget et al., 2019). In particular, the role of the SASP *in vivo* and in human disease is poorly understood, partly because of a paucity of suitable models and experimental settings allowing the study of SASP expression and how it affects early malignant progression. We therefore utilized our early lung cancer GEMM as well as our human early NSCLC samples to investigate the role of the TLR2-regulated SASP in this context. We performed IHC staining of key TLR2-regulated SASP factors, including interleukin-1-alpha (IL-1*α*), interleukin-1-beta (IL-1*β*), and the TLR2 damage-associated molecular pattern (DAMP) acute phase serum amyloid A (A-SAA) in normal alveolar tissue and WT and *Tlr2*^−/−^ lung tumors from Kras^LSL–G12D/+^ mice. We found that SASP expression is induced in WT lung tumors, but *Tlr2*^−/−^ lung tumors had significantly reduced expression of all three SASP factors (Figure 3). When we repeated this staining in KP lung tumors, we again saw significantly reduced expression in *Tlr2*^−/−^ samples (Figure S2C), confirming that *Tlr2* activates proinflammatory SASP signaling independent of *Trp53*.

The SASP mediates recruitment of immune cells (predominantly those of the monocyte/macrophage lineage) that clear senescent cells (Kang et al., 2011; Xue et al., 2007). Therefore, we wanted to assess whether impairment of Tlr2-SASP expression impaired recruitment of immune cells to lung tumors. To do this, we performed flow cytometry analysis on single-cell suspensions from tumor-bearing lungs from *Kras*^*LSL-G12D*/+^;*Tlr2*−/− and control mice. We saw a significant reduction in total immune cells (CD45^+^) in *Tlr2*^−/−^ tumor-bearing lungs (Figure 3C), and this was primarily driven by a reduction in alveolar macrophages (SiglecF^+^CD11c^+^) and monocytes (SiglecF^−^CD11b^+^Ly6C^+^), but the reduction in monocytes did not reach statistical significance (Figures 3B and 3C). We then investigated tumor infiltration of monocyte/macrophages using IHC staining for the monocyte/macrophage marker CD68 and found significantly reduced infiltration of CD68^+^ cells in *Tlr2*^−/−^ tumors (Figures 3D and 3E). Local delivery of the recombinant SASP factors (rIL-1*α* and rIL-1*β*) into the lungs of *Tlr2*^−/−^ mice induced a robust increase in CD68^+^ cells in the lungs compared with controls (Figure S2D), confirming that *Tlr2*^−/−^ monocyte/macrophages are capable of responding to local SASP factors. Local delivery of rIL-1*α* and rIL-1*β* into the lungs of *Tlr2*^+/+^ mice recapitulated an effect similar to that observed in *Tlr2*^−/−^ mice (Figure S2D), confirming that there is no blunted immune response to local SASP factors in *Tlr2*^−/−^ mice. To investigate whether the effect of *Tlr2* loss was intrinsic to epithelial cells or due to global (principally immune cell) *Tlr2* loss. We performed *in vivo* somatic genome editing using CRISPR-Cas9 to assess the effect of *Tlr2* deletion in epithelial cells only. We used the pSECC lentivirus system (Sanchez-Rivera et al., 2014), which permits co-expression of Cre-recombinase and CRISPR-Cas9 machinery, allowing deletion of *Tlr2* and concurrent activation of oncogenic *Kras*^*G12D*^ signaling in lung epithelial cells alone (Figure S2E). The efficacy of our *Tlr2* guide RNA (gTlr2) expressing pSECC virus was assessed *in vitro* by infecting mouse embryonic fibroblasts (MEFs) with a control (gTomato) or gTlr2-pSECC lentivirus and measuring *Tlr2* RNA and protein expression via qRT-PCR and western blot, respectively (Figures S2F and S2G). *In vivo* assessment of pSECC efficacy was performed using RNA *in situ* hybridization (RNA-ISH), which demonstrated a clear reduction in *Tlr2* transcript expression in gTlr2-targeted tumors (Figure S2H). *Kras*^*LSL-G12D*/+^ mice were inoculated with a gTomato- or gTlr2-expressing pSECC lentivirus, and lung tissue was harvested for IHC analysis. We saw a non-significant increase in expression of the proliferation marker Ki67 in *Tlr2*-targeted tumors (p = 0.051; Figure S2I). In concert with this, we saw that gTlr2-targeted tumors expressed significantly lower levels of SASP factors and had significantly fewer recruited monocytes/macrophages than gTomato-targeted tumors (Figures S2I and S2J). These data suggest that epithelial Tlr2-SASP expression controls recruitment of monocyte/macrophages to lung tumors. We did not observe a significant difference in lymphoid cell recruitment in *Tlr2*^−/−^ tumor-bearing lungs via flow cytometry analysis (Figures S3A and S3B), and this was confirmed using IHC staining for the T cell markers CD3, CD4, and CD8 (Figures S3C and S3D). Our flow cytometry experiments included analysis of γδ T cells, a population of lung-resident innate lymphoid cells that have been shown to promote lung tumorigenesis in response to altered lung commensal microbiota (Jin et al., 2019). We observed no significant increase in γδ T cells in *Tlr2*^−/−^ tumor-bearing lungs, suggesting that the tumor-promoting effect of *Tlr2* loss is not mediated via γδ T cell expansion (Figures S3A and S3B).

To further investigate the functional relevance of the impaired immune response after *Tlr2* loss, we took advantage of a well-characterized model of senescence surveillance, where oncogenes are expressed in murine hepatocytes via hydrodynamic tail vein delivery of transposable elements (Carlson et al., 2005; Kang et al., 2011). Hydrodynamic delivery of an oncogenic *Nras*-expressing plasmid (*Nras*^G12V^-GFP) was performed in WT and *Tlr2*^−/−^ mice. An effector loop mutant incapable of downstream oncogenic signaling (*Nras*^*G12V/D38A*^-GFP) served as a negative control. Six days after delivery, expression of the active oncogene construct resulted in a robust immune infiltrate consisting mainly of monocyte/macrophages (F4/80^+^ cells) that surrounded *Nras*^G12V^-expressing hepatocytes, but this was markedly impaired in *Tlr2*^−/−^ mice (Figure S3E). This impaired response corresponded with a significant impairment in clearance of senescent *Nras*^G12V^-expressing hepatocytes at 12 days (Figure S3F). These results suggest that Tlr2 controls recruitment of myeloid cells to tumors, which may mediate immune surveillance during tumor initiation.

We then studied the role of the TLR2-regulated SASP in human lung cancer. We performed IHC staining for TLR2 and IL-1*β* on consecutive sections from human LUAD samples. Not only did we observe significantly increased IL-1*β* expression in LUAD epithelium compared with normal epithelium (Figures 3F and S3G), but we observed a striking overlap of TLR2 and IL-1*β* expression (Figure 3F). We then performed automated H-score analysis for TLR2 and IL-1*β* on identical tumor regions from consecutively stained sections and found a significant positive correlation between TLR2 and IL-1*β* expression (Figure 3G), suggesting that TLR2 may regulate expression of the SASP in LUAD epithelium. We also performed IHC staining on serial sections from preinvasive LUSC samples and again observed that not only is IL-1*β* expression increased in regressive lesions compared with progressive lesions (Figures 3H and S3H), but this expression is highly epithelial in origin and significantly correlated with TLR2 expression (Figures 3H and 3I). We also analyzed gene expression of the TLR2-regulated SASP (identified previously; Hari et al., 2019) in preinvasive LUSC lesions and found that expression of the TLR2-SASP signature is significantly increased in lesions that subsequently regress to normal epithelium (Figures 3J and 3K). Furthermore, expression of the TLR2-SASP significantly correlated with *TLR2* expression (Figure 3L). Taken together, these data suggest that TLR2 regulates a proinflammatory SASP in NSCLC correlating with impairment of tumor progression.

### Pharmacological intervention with Tlr2 agonists prevents early lung tumor growth

**Figure 4.**
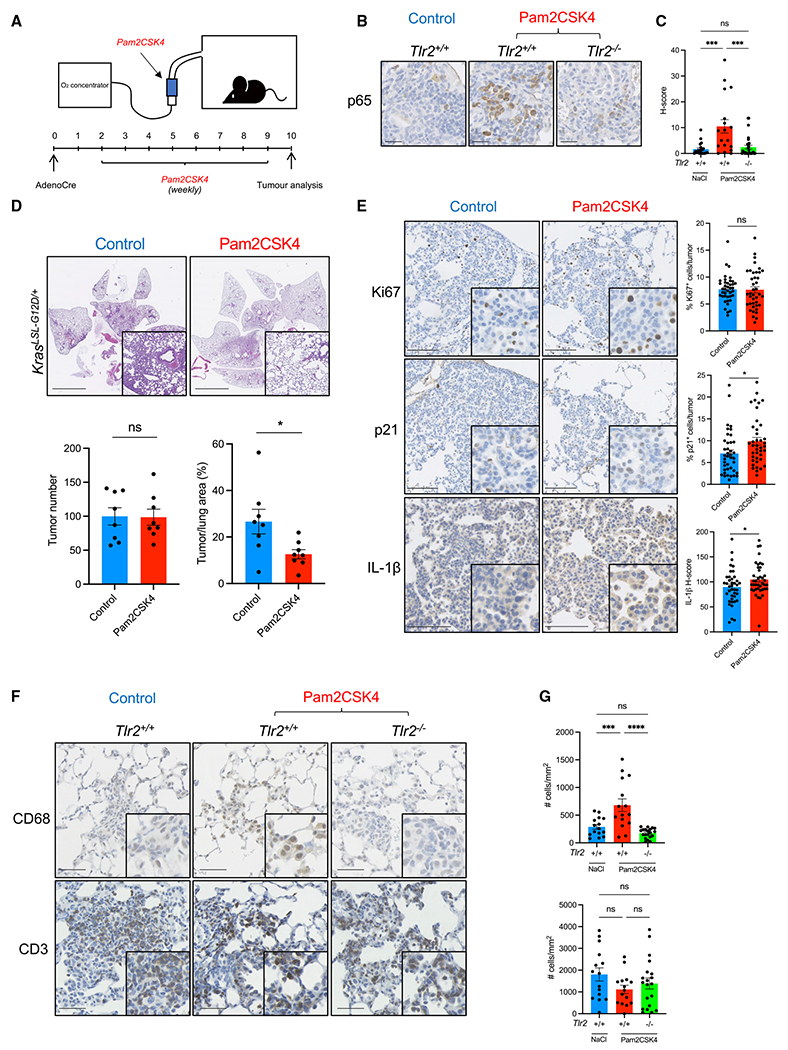
TLR2 activation inhibits lung tumor growth (A) Kras^LSL-G12D/+^ mice were inoculated with AdenoCre as described previously. Two weeks after inoculation, they were subjected to a weekly nebulized dosing with Pam2CSK4 or control (0.9% NaCl solution) for 8 weeks prior to sacrifice and histological analysis. (B and C) Representative IHC staining for p65 (RelA) in early lung tumors from Tlr2 WT (*Tlr2*^+/+^) or *Tlr2*^−/−^ 1 h after treatment with Pam2CSK4 or control (NaCl)(B) with corresponding quantification (C). Data presented as mean +/− SEM. Statistical analysis was performed using a one-way ANOVA with post hoc Tukey testing for multiple comparisons. ***p < 0.001. Scale bars, 25 μm. (D) Representative H&E staining of lung sections from Kras^LSL-G12D/+^ mice after control or Pam2CSK4 treatment with corresponding tumor number and tumor burden analysis. n = 8 mice per group. Data presented as mean +/− SEM. Statistical analysis was performed using Student’s t test. * *p < 0.05. Scale bars, 5 mm. (E) Representative IHC staining for Ki67, p21, and IL-1*β* in lung tumors from Kras^LSL-G12D/+^ mice after control or Pam2CSK4 treatment with corresponding quantification. n = 8 mice per group (five tumors per mouse analyzed). Data presented as mean +/− SEM. Statistical analysis was performed using Student’s t test. *p < 0.05. Scale bars, 100 μm. (F and G) Representative IHC staining for CD68 and CD3 in early lung tumors from Tlr2 WT (*Tlr2*^+/+^) or *Tlr2*^−/−^ 1 h after treatment with Pam2CSK4 or control (NaCl) (F) with corresponding quantification (G). Data presented as mean +/− SEM. Statistical analysis was performed using a one-way ANOVA with post hoc Tukey testing for multiple comparisons. ***p < 0.001, ****p < 0.0001. Scale bars, 50 μm.

We then wanted to assess, in a pre-clinical model, whether activation of Tlr2 signaling could be harnessed therapeutically. We used the synthetic Tlr2 agonist Pam2CSK4, delivered via nebu-lization, to allow direct repeated administration of drug to the lung epithelium. Kras^LSL–G12D/+^ mice on a *Tlr2*^+/+^ background were inoculated intranasally with AdenoCre as described previously. Two weeks later, they were subjected to weekly dosing with nebulized Pam2CSK4 or vehicle control (0.9% sodium chloride) for 8 weeks prior to sacrifice and lung tumor analysis (Figure 4A). Activation of Tlr2 signaling was confirmed with IHC staining for p65 (RelA), a key component of the NF-κB pathway (Figures 4B and 4C). After repeated Pam2CSK4 treatment, the tumor burden was significantly reduced in comparison with controls (Figure 4D). This was associated with significantly increased p21 expression, but Ki67 expression was unchanged (Figure 4E). We analyzed expression of the SASP factors IL-1 *α*, IL-1*β*, and A-SAA in tumors after Pam2CSK4 treatment and found that this was increased, but that of IL-1*α* did not reach statistical significance (Figures 4E and S4A), indicating increased SASP expression after Tlr2 activation. We then analyzed immune cell recruitment to early tumors after treatment with Pam2CSK4 and found that CD68^+^ cell recruitment to *Tlr2*-proficient tumors is significantly increased after agonist treatment, but CD3^+^ cell recruitment was unchanged (Figures 4F and 4G). This suggests a higher contribution to senescence immune surveillance after treatment with a Tlr2 agonist rather than direct effects on proliferation. We repeated this experiment in *Tlr2*^−/−^ mice and saw no significant difference in tumor burden, Ki67 or SASP expression (Figure S4B), confirming that Pam2CSK4 acts via Tlr2 to mediate its anti-tumor response. Our data show that activation of Tlr2 can inhibit early lung tumor growth, highlighting TLR2 as a therapeutic target for treatment of early-stage NSCLC. Mice treated with Pam2CSK4 exhibited no observable side effects or altered phenotype, further suggesting TLR2 as a possible drug target in early lung cancer.

## DISCUSSION

Our report demonstrates that *TLR2* has a tumor suppressor function in NSCLC. In a unique cohort of preinvasive and early-invasive human NSCLC samples, we show that *TLR2* is highly expressed in tumor epithelium and correlates with improved survival and clinical regression. Correlation between *TLR2* expression and improved clinical outcomes in lung cancer has also been reported previously in a pan-TLR expression analysis (Bauer et al., 2017) and our data support this finding. Further studies using a *Kras*^*G12D*^ driven GEMM of NSCLC suggest that p53-p21 signaling is integral to the tumor suppressor response elicited by Tlr2. However, by utilizing co-existent conditional *Kras*^*G12D*^ activation and *Trp53* KO models, we show that this tumor suppressor role is not entirely dependent on this cell-intrinsic cell cycle arrest pathway because heightened tumor burden and poorer survival were observed in *Tlr2*^−/−^ mice regardless of *Trp53* status. We therefore sought to determine whether cell-extrinsic mechanisms such as SASP expression contribute to the tumor suppressor effect of *Tlr2*. The SASP has wide-ranging effects, including pro- and anti-tumor responses (Coppe et al., 2010). Studies describing the pro- and anti-tumorigenic effects of the SASP have mainly highlighted the role of the SASP *in vitro* or in mouse models involving subcutaneous co-implantation of senescent fibroblasts with transformed cell lines. Other work in liver models of exogenous oncogene activation has shed light on the role of immune surveillance in premalignancy (Kang et al., 2011; Xue et al., 2007), but, to date, no studies have robustly shown an anti-tumor role of SASP signaling in human cancer. We first investigated SASP expression in our GEMM lung tumors and found that expression of the SASP is significantly impaired in *Tlr2*-null tumors (with and without active Trp53), suggesting that SASP expression may explain the Trp53 independent tumor suppressor effects of Tlr2. Suppression of p16 has been shown recently to perturb expression of the SASP after oncogene activation (Buj et al., 2021), suggesting that intact p16-Rb signaling in our *Trp53*-null tumors may maintain SASP expression. We observed significantly impaired p16 expression in *Tlr2*^−/−^ KP lung tumors. It has also been shown that mutant *TRP53* interferes with cGAS-STING-TBK1 signaling, subsequently impairing the innate immune response and promoting cancer progression (Ghosh et al., 2021). This effect is not observed in *Trp53*-null cells, suggesting that mutant *TRP53* performs gain-of-function activities to inactivate tumor immune surveillance. cGAS-STING activation is a key event in expression of the SASP, mediated via cytoplasmic chromatin sensing (Dou et al., 2017; Gluck et al., 2017), and TLR2 functions downstream of this activation (Hari et al., 2019). Therefore, it remains to be determined whether intact cGAS-STING activation may explain the persistent effect of *Tlr2* loss we observe in our *Trp53*-null tumors.

We then analyzed SASP expression in human NSCLC samples and observed a striking correlation with TLR2 expression in LUAD and LUSC and a significant correlation with clinical regression in preinvasive LUSC. Gene expression profiling of human preinvasive LUSC lesions has revealed an abundance of immune sensing during the early stages of tumorigenesis with activated T cells and myeloid cells (including macrophages), with immune escape mechanisms activated prior to invasion (Mascaux et al., 2019). Supporting this, we have shown previously that immune surveillance is strongly implicated in preinvasive LUSC lesion regression (Pennycuick et al., 2020), leading us to suggest that this may be supported by the *TLR2*-regulated SASP. More recently, whole-exome sequencing and gene expression analysis have also identified immune escape mechanisms as key events in preinvasive LUAD lesions, suggesting a key role of immune surveillance in NSCLC progression (Altorki et al., 2022). Macrophages make up the majority of the tumor immune infiltrate in human lung cancer (Conway et al., 2016), and studies examining the microanatomical location of macrophages have revealed that high macrophage infiltration within lung tumor epithelium (versus tumor stroma) is significantly associated with improved patient survival (Dai et al., 2010; Kim et al., 2008; Welsh et al., 2005). Although our report does not show evidence that TLR2-regulated proinflammatory SASP signaling mediates lesion regression in LUAD (because these lesions cannot be tracked longitudinally), it is tempting to speculate that activated TLR2-SASP signaling in lung tumors instructs recruitment of macrophages to the epithelium to subsequently impair tumor progression via immune surveillance. Senescence surveillance, described in liver models of exogenous oncogene activation, is an adaptive immune response orchestrated by CD4^+^ T cells, but the effector cells are recruited monocytes/macrophages (Kang et al., 2011). However, whether the same mechanism occurs in the lung remains to be seen. Corresponding with the reduction in SASP expression in our *Tlr2*-null GEMM lung tumors, we identified a significant reduction in total and tumor-specific myeloid cells in tumor-bearing lungs from *Kras*^*LSL-G12D*/+^;*Tlr2*^−/−^ mice. We observed no changes in T cell recruitment/infiltration, but tumors from *Kras^G12D^*-driven GEMMs of lung cancer have few protein-altering mutations and are therefore unlikely to express significant numbers of neoantigens (McFadden et al., 2016). Therefore, because of the limitation of our GEMM, we cannot exclude heightened T cell-mediated immunity via the Tlr2-regulated SASP as a driver of lesion regression.

Last, to determine whether our findings have therapeutic potential, we tested whether activation of Tlr2 could perturb lung tumor growth. We found that, after inhalational delivery of a synthetic Tlr2 agonist, lung tumor growth was significantly reduced, and this corresponded with increased expression of p21, key SASP factors, and recruited myeloid cells. Several studies have highlighted possible tumor-promoting effects of the SASP (Bavik et al., 2006; Coppe et al., 2008; Krtolica et al., 2001). However, our data argue against this and highlight that increased proinflammatory SASP expression (via Tlr2 activation) in early lung tumors can impair tumor progression. There are many ongoing efforts to design ways of inhibiting the SASP as a cancer therapy, but our data urge caution against this approach. Inhibiting the SASP in established malignant tumors, where it contributes to chronic inflammation and tumor growth, would be beneficial, but our data suggest that inhibition of SASP expression in premalignant contexts could be deleterious. TLR2 agonists have been studied previously in a randomized clinical trial in combination with chemotherapy in advanced metastatic NSCLC (Belani et al., 2017). However, no survival benefit was observed. This is likely explained by the reduced TLR2 expression in advanced LUAD lesions, as demonstrated by our data. The pro-senescent and pro-inflammatory effects of chemotherapy likely reduce the effect of specific TLR2 activation. It could therefore be informative to determine the effect of Tlr2 agonists in combination with a chemotherapy agent in our GEMMs. However, we propose that targeting TLR2 signaling therapeutically in early-stage/preinvasive contexts (where chemotherapy is not the first-line treatment) could be a viable strategy to impair malignant progression. This approach would require identification of patients with a high preinvasive disease burden, perhaps via circulating SASP biomarkers.

### Limitations of the study

First, we observed reduced infiltration of myeloid populations in lung tumors after *Tlr2* loss, and showed, using exogenous oncogene activation in the liver, that this leads to impaired immune clearance of premalignant cells. However, we have not shown direct evidence that impaired recruitment of myeloid cells to lung tumors underlies the tumor-promoting effects of *Tlr2* loss in this context. Future studies that are not within the scope of this report, involving specific immune cell depletion, will be required to determine whether myeloid subpopulations are integral to this process in the lungs. Second, we showed that Tlr2 may be a potential therapeutic target in early lung cancer. However, data supporting this involves GEMMs of lung cancer, which do not fully capture the genetic heterogeneity of human disease; therefore, the effect of TLR2 activation in human cancer may not be as robust.

## STAR*METHODS

### RESOURCE AVAILABILITY

#### Lead contact

Further information and requests for resources or reagents should be directed to Fraser Millar (fraser.millar@ed.ac.uk).

#### Materials availability

This study did not generate new unique reagents.

#### Data and code availability

- This paper analyses existing, publicly available data. The references for these datasets are listed within the relevant results sections.
- This study does not report original code.
- Any additional information required to reanalyse the data reported in this paper is available from the lead contact upon request.

### EXPERIMENTAL MODEL AND SUBJECT DETAILS

The mouse strains used in this study are described in the animal experiments section below and the key resources table.

Details regarding the human samples used in this study are described in the human lung cancer sample analysis section below and the key resources table.

### METHOD DETAILS

#### Animal experiments

All mice were housed in a specific pathogen free environment with food and water *ad libitum* in accordance with UK home office guidance at the Biomedical Research Facility, University of Edinburgh. Black six (C57BL/6) mice harboring the conditional oncogenic *Kras*^*G12D*^ allele (Kras^LSL–G12D/+^) were purchased from the Jackson Laboratory (jax.org) and interbred with black six (C57BL/6) mice lacking *Tlr2* (*Tlr2*^−/−^) (received from Dr Jen Morton, University of Glasgow) to generate our *Kras*^*LSL-G12D*/+^;*Tlr2*^−/−^ line. This line was then interbred with black six (C57BL/6) mice harboring the ‘floxed’ *Trp53* allele (*Trp53*^*fl/fl*^) (received from Dr Luke Boulter, University of Edinburgh) to generate our *Kras* ^*LSL-G12D*/+^;*Trp53*^*fl/fl*^;*Tlr2*^−/−^ line. Male and female mice between the ages of 8–12 weeks were anesthetized with medetomidine and ketamine prior to intranasal inoculation with 40ul of virus solution (1.5 × 10^∧^7 plaque-forming units (PFU) of adenovirus expressing Cre-recombinase under control of the CMV promoter suspended in minimum essential media (MEM). Mice were humanely sacrificed 8–12 weeks later. Lung tissue was harvested, inflated with 10% neutral buffered formalin (NBF) and fixed in NBF overnight prior to tissue processing and paraffin embedding. For recombinant IL1α/IL1β inoculation, mice were anesthetized as described above and intranasally inoculated with 100ng of carrier free recombinant protein (rIL-1*α* – Biolegend #575002, rIL-1*β* – Biolegend #575102) suspended in sterile phosphate buffered saline (PBS). For Tlr2 agonist experiments, 100ug Pam2CSK4 was diluted in 3mLs 0.9% NaCl and delivered via a nebuliser to mice housed in a nebuliser chamber over 30 min. Control mice received 0.9% NaCl only. This was performed weekly for eight weeks prior to humane sacrifice and tissue processing as above. For hydrodynamic tail vein injection experiments, wild-type (WT) and Tlr2 null (*Tlr2*^−/−^) mice on a C57BL/6 background aged between 6 and 12 weeks were included in the study. DNA plasmids were prepared using the Qiagen Plasmid Maxi Kit (Qiagen, Germany) as per the manufacturer’s instructions. Each mouse received 6mg of a sleeping beauty transposase expressing plasmid (CMV-SB13 transposase) and 20μg of transposon (pT3-Nras^G12V^-IRES-GFP/Luc or pT3-Nras^G12V/D38A^-IRES-GFP) encoding plasmid diluted in 0.9% NaCl solution to 10% of the animal’s body weight delivered via the lateral tail vein within 10 seconds.

#### Immunohistochemistry

3μm sections were cut onto adhesion slides from NBF fixed, paraffin embedded (FFPE) samples then placed in a 60°C oven for at least two hours. Slides were de-waxed in xylene and rehydrated in ethanol of decreasing concentrations. Antigen retrieval was performed using heated sodium citrate buffer for ten minutes or proteinase K. Samples were blocked with hydrogen peroxide, avidin/biotin block (if using biotinylated secondary antibodies) and protein block prior to incubation with primary antibody (see key resources table). Samples were washed then incubated with appropriate biotinylated secondary antibodies followed by streptavidin-HRP conjugate (VECTASTAIN ABC reagent) then revealed with 3,3’Diaminobenzidine (DAB) diluted in DAB substrate (Abcam), with the exception of CD8 staining which was performed using HRP conjugated secondary antibodies followed by revealing with DAB. Where mouse primary antibodies were used, a mouse on mouse blocking kit was used for blocking and antibody incubation steps (M.O.M^®^ Immunodetection kit, Vector Labs). Samples were counterstained with haematoxylin before dehydration with ethanol, clearing with xylene and mounting. Immunohistochemistry slides were imaged using the Hamamatsu Nanozoomer XR microscope with NDP scan v3.1 software. Tumor burden quantification was performed in a blinded fashion on haematoxylin and eosin (H&E) stained slides using NDP viewerv2 software. Immunohistochemistry quantification (positive cell detection or H-score analysis) was performed using QuPath software v0.1.2 (Bankhead et al., 2017). Histological grade analysis was performed in a blinded fashion using predefined criteria (DuPage et al., 2009) and validated by an independent reviewer on a subset of tumors (n = 120) with strong inter-rater agreement as assessed using Cohen’s kappa coefficient (weighted Kappa 0.854, unweighted kappa 0.789 (95% CI 0.698–0.880)). Co-immunofluorescence staining was performed on 3μm FFPE samples that were dewaxed and rehydrated as above. Permeabilization was performed with a 0.1 % Triton X-100 solution in PBS prior to incubation with primary antibodies. Samples were then washed followed by incubation with appropriate fluo-rophore conjugated secondary antibodies. Samples were then washed, stained with DAPI and mounted with Vectashield^®^. Samples were imaged using a confocal microscope using NIS-Elements software (Nikon).

#### Total RNA preparation and quantitative reverse transcription polymerase chain reaction (qRT-PCR)

Murine lung tissue was dissected, roughly diced and homogenized with trizole. Choloform was added for phase separation and RNA was precipitated using isopropanolol. RNA wash and elution was performed thereafter using the Qiagen RNeasy plus mini kit (Qiagen, Germany), according to the manufacturer’s instructions. cDNA was synthesized using qScript cDNA SuperMix (Quanta Biosciences) from 1ug of RNA in a 40ul reaction. qRT-PCR was performed using 1ul of cDNA per reaction well and SYBR Select Master Mix (Life Technologies) using 200nM of forward and reverse primers in 10ul. Samples were run in triplicate on a StepOnePlus Cycler (Thermo Fisher Scientific). mRNA expression was analyzed by the delta Ct method and normalized to levels of beta actin mRNA expression. All primers are listed in Table S1.

#### Protein extraction and western blot analysis

Murine lung tissue was dissected, roughly diced and homogenized using a Dounce homogenizer in 500ul of Sodium dodecyl sulfate (SDS) lysis buffer on ice (4% SDS, 150mM NaCl, 50mM Tris-HCl pH 7.5). Samples were incubated on ice for 10 minutes prior to centrifugation at >13,000rpm for 10 min at 4°C. The supernatant was removed and stored at −20°C. Supernatants were quantified using a NanoDrop (Thermo Fisher). Samples were prepared for loading by mixing with Laemmli sample buffer and incubating at 95°C for 5 minutes. Protein samples were resolved by polyacrylamide gel electrophoresis (150V for 1 h) and transferred onto a nitrocellulose membrane (200mA for 2 h). Membranes were blocked for 1 h in 5% milk/tris-buffered saline – 0.1% Tween (TBST). Indicated primary antibodies (see key resources table) were diluted in 5% milk/TBST (or 2.5% BSA/TBST for alpha tubulin) and incubated overnight at 4°C. Blots were washed three times in TBST for more than 30 minutes. Membranes were then incubated in secondary antibodies prepared in 5% milk/TBST for 1 hour at room temperature. Membranes were washed as before, prior to application of enhanced chemical luminescence (Amersham) detection reagent and imaging.

#### Flow cytometry

Tumor bearing mice were humanely sacrificed ten weeks after inoculation with Cre-expressing adenovirus. The lung vasculature was perfused with up to 30mLs of ice-cold PBS over 30 seconds via the right ventricle to flush the lungs of all blood. Tissue was collected and weighed, roughly diced prior toa 25 minute incubation with 10mLs of a digestion enzyme mix (0.8mg/mL collagenase V, 0.625mg/mL collagenase D, 1mg/mL dispase and 30μg/mL DNase) in a shaking incubator at 37°C. Digested lung tissue was filtered through a 100μm cell strainer, washed twice with RPMI (Lonza) and incubated with 3mLs red cell lysis buffer (Sigma) for three minutes. After further washing samples were filtered through a 35μm cell strainer directly into 5mL (12 × 75) polystyrene round-bottom FACS tubes and counted using a Muse Cell Analyser (Merck Millipore). Single cell suspensions were blocked with 10% mouse serum and 1% Fc block prior to incubation with cell surface fluorophore conjugated primary antibodies (see key resources table). Samples were analyzed on a BD LSR Fortessa X-20 analyser using BD FACSDiva 8.0.1 software. Data were analyzed using FloJo software v10.2. Gating was designed using fluorescence minus one (FMO) samples. Compensation was performed using single antibody stained OneComp eBeads (ThermoFisher). Total cell number was determined by multiplying the percentage of live fluorophore positive cells by the total number of live cells isolated per sample and this was normalised to tissue weight.

#### Molecular cloning and lentivirus generation

Empty vector pSECC plasmids were obtained from Tyler Jacks via Addgene (#60820). Guide RNA (gRNA) oligos were selected from the GeCKO library (Shalem et al., 2014) and ordered from Sigma. pSECC vectors were digested with BsmBI restriction enzymes and ligated with compatible annealed oligos (Table S2). Lentiviruses were produced by polyethylenimine (PEI) mediated co-transfection of 293T cells with lentiviral backbone and packaging plasmids (PAX2 and VSVG) with pSECC plasmids. Supernatant was collected at 48- and 72-hours post transfection and virus was concentrated using Lenti-X^™^ concentrator (Takara) and resuspended in appropriate volumes of MEM. Lentiviral titrations were performed by infecting 3TZ cells (containing the loxP-STOP-loxP β-Gal cassette) followed by beta-galactosidase staining. Control (gTomato) and *Tlr2* targetting pSECC (gTlr2) lentivirus solutions of equal concentration were administered intranasally to Kras^LSL–G12D/+^ mice as described above. Lung tissue was harvested and processed as described above.

#### RNA *in situ* hydridization

The methods used for RNA *in situ* hybridization (RNA-ISH) were as described recently (Fernandez-Guerrero et al., 2021). 7μm sections of FFPE lung samples from pSECC treated mice were cut onto adhesion slides. To ensure identical treatment, one control sample (gTomato) and one *Tlr2* targetted sample (gTlr2) were placed side by side in the same staining chamber. After dewaxing and rehydration, tissue sections were permeabilized by incubation with proteinase K (10μg/mL) for 7.5 minutes at room temperature (RT), then washed in PBS twice and post-fixed with 4% paraformaldehyde (PFA) for 20 minutes. An acetylation step (250mL 0.1 M triethanolamine, 625μL 0.066 mM acetic anhydride, 10 minutes) was performed to reduce background signal. Hybridization for *Tlr2* was performed using an *in vitro* transcribed digoxigenin-labelled RNA probe overnight (ON) at 65°C in a humidified chamber. Next day, post-hybridization washes (50% formamide/50% 1x saline sodium citrate (SSC) 30 minutes; 2 x SSC 20 minutes; 0.2 x SSC twice 20 minutes) at 65°C were performed to remove unspecific binding. Samples were subjected to two washes in maleic acid buffer containing Tween 20 (MABT) buffer at RT, and then blocking (20% sheep serum in MABT, pH 7.5) for 1 hour at RT, before an incubation ON with anti-digoxigenin antibody (1/2500 in 5% sheep serum/MABT) at 4°C. Sections were washed three times in MABT buffer 5 minutes at RT and once in NTM, pH 9.5 for 10 minutes. The detection reaction was performed with NBT (3 μL/mL)/BCIP(2.3 μL/mL) in NTM, pH 9.5. When the desired signal level was reached, the reaction was stopped with two PBS washes and fixed in 4% PFA for 20 minutes. After dehydration and mounting steps, slides were digitalized with an Axio Scan.Z1 slide scanner (Zeiss) using an x40 objective in brightfield. Resources used for RNA-ISH are outlined in the key resources table and Table S3. *Tlr2* transcript signal was scored on control and *Tlr2* targetted tumors as described in the original study describing pSECC (Sanchez-Rivera et al., 2014).

#### Human lung cancer sample analysis

Anonymous human LUAD samples were obtained with written informed consent and ethical approval from the NRS Lothian Bioresource, Edinburgh. Human preinvasive LUSC samples were obtained with written informed consent from patients enrolled in the University College London Hospital (UCLH) surveillance study and ethical approval was provided by the UCL/UCLH local ethics committee. LUAD and LUSC samples were stained and analyzed as described above. Protein expression correlation was performed on serial sections using QuPath (Bankhead et al., 2017). All human gene expression data presented were re-analysed from published datasets and sample acquisition and analysis has been previously described (Chen et al., 2019; Teixeira et al., 2019). Briefly, RNA-sequencing data from LUAD lesions were downloaded with permission from the European Genome-Phenome Archive (EGA) and aligned to the human genome (GRCh38) using STAR (v2.6.1) (Dobin et al., 2013) and read counts were quantified using Salmon (version 1.4.0). Differential gene expression analysis was performed using the DESeq2 package on R (version 4.0.3) (Love et al., 2014). Exome sequencing data from the same LUAD samples (Chen et al., 2019) were used to analyze LOH frequency. The variant caller VarDict Java (1.8.3) was used to call variants with an allele frequency >0.1 in the tumor exome samples against the GRCh37 human reference genome. The R (4.2.0) package DNACopy (1.70) was used to call regions of LOH in each sample using binarized homozygous and heterozygous variant calls. Regions of LOH that overlapped the *TLR2* locus were extracted and the mean score of the locus was compared between preinvasive and invasive samples.

For progressive vs regressive preinvasive LUSC gene expression analysis, Illumina and Affymetrix microarray platforms were used to analyze RNA extracted from patient samples from the UCLH Surveillance study. ‘Index’ biopsies were used for gene expression analysis and were defined as a preinvasive LUSC biopsy sample of equal grade that was performed four months prior to either a diagnostic cancer biopsy (progression) or a normal/low grade lesion biopsy (regression). Data from preinvasive LUSC gene expression were analyzed using R (version 4.0.3) using a linear mixed effects model to account for multiple samples per patient. The TLR2-SASP signature signature was determined by calculating the geometric mean of gene expression values of each of the SASP factors regulated by TLR2 as described previously (Hari et al., 2019).

Tumor and adjacent normal tissue methylation data from the TCGA (Cancer Genome Atlas Research, 2012,^2014^) was downloaded and analyzed using the Chip Analysis Methylation Pipeline (ChAMP) Bioconductor package with default settings (Morris et al., 2014).

### QUANTIFICATION AND STATISTICAL ANALYSIS

Statistical analysis was performed using R (version 4.0.3) and GraphPad Prism software (version 9.0.1). Details regarding the statistical tests used can be found in the figure legends. The statistical tests used were justified as appropriate based on sample size and distribution. Student’s t test or Mann-Whitney tests (or equivalent tests for paired analysis) were used for two-condition comparisons, with a significance cut off of p < 0.05.

## Supplementary Material

supplemental data

## Figures and Tables

**Table:** KEY RESOURCES TABLE

REAGENT or RESOURCE	SOURCE	IDENTIFIER
Antibodies		
Rabbit monoclonal anti-Ki67	Abcam	Cat #Ab16667; RRID:AB_302459
Mouse anti-p21	BD biosciences	Cat #556431; RRID:AB_396415
Rat anti-p19/Arf	Abcam	Cat #Ab174939; RRID:AB_2924683
Mouse anti-p16	Abcam	Cat #Ab54210; RRID:AB_881819
Goat anti-IL-1 *α*	R&D systems	Cat #AF-400; RRID:AB_354473
Rabbit anti-IL-1 *β*	Santa Cruz	Cat #sc-7884; RRID:AB_2124476
Rabbit anti-SAA1	Biorbyt	Cat #orb228668; RRID:AB_2924684
Mouse anti-Nras	Santa Cruz	Cat #sc-31; RRID:AB_628041
Rat anti-F4/80	Abcam	Cat #Ab6640; RRID:AB_1140040
Rabbit anti-CD3	Abcam	Cat #Ab5690; RRID:AB_305055
Rabbit anti-CD4	Abcam	Cat #Ab183685; RRID:AB_2686917
Rabbit anti-CD8	Abcam	Cat #Ab217344; RRID:AB_2890649
Rabbit anti-CD68	Abcam	Cat #Ab125212; RRID:AB_10975465
Rabbit anti-TLR2	Novus Bio	Cat #NBP2-24861; RRID:AB_2924685
Mouse anti-Human IL1B	Santa Crus	Cat #sc-32294; RRID:AB_627790
Mouse anti-NF-κB p65	Proteintech	Cat #66535-1-Ig; RRID:AB_2881898
Biotinylated goat anti-mouse IgG	Vector labs	Cat #BA9200; RRID:AB_2336171
Biotinylated goat anti-rabbit IgG	Vector labs	Cat #BA1000; RRID:AB_2313606
Biotinylated rabbit anti-goat IgG	Vector labs	Cat #BA5000; RRID:AB_2336126
Anti-rabbit HRP polymer	Dako	Cat #K4003; RRID:AB_2630375
Goat anti-mouse AF488	ThermoFisher	Cat #A11029; RRID:AB_2534088
Goat anti-rat AF594	ThermoFisher	Cat #A11007; RRID:AB_10561522
Mouse monoclonal anti-*α*-tubulin	Sigma	Cat #T9026; RRID:AB_477593
Anti-rabbit IgG HRP-linked	CST	Cat #7074S; RRID:AB_2099233
Anti-mouse IgG HRP-linked	CST	Cat #7076S; RRID:AB_330924
Anti-mouse CD45 (Brilliant Violet 421)	Biolegend	Cat #103133; RRID:AB_10899570
Anti-mouse CD3 (PE/Cyanine7)	Biolegend	Cat #100219; RRID:AB_1732068
Anti-mouse CD4 (PE)	Biolegend	Cat #100407; RRID:AB_312692
Anti-mouse CD8 (Super bright 600)	Invitrogen	Cat #63-0081-80; RRID:AB_2637162
Anti-mouse TCR γδ (Alexa Fluor^®^ 488)	Biolegend	Cat #118127; RRID:AB_2562770
Anti-mouse/human CD11b (APC)	Biolegend	Cat #101211; RRID:AB_312794
Anti-mouse CD11c (APC/Cyanine7)	Biolegend	Cat #117323; RRID:AB_830646
Anti-mouse Siglec-F (PE)	Biolegend	Cat #155505; RRID:AB_2750234
Anti-mouse Ly6C (FITC)	Biolegend	Cat #128005; RRID:AB_1186134
Anti-mouse Ly6G (Alexa Fluor^®^ 700)	Biolegend	Cat #127621; RRID:AB_10640452
Anti-mouse CD19 (PE/Cyanine7)	Biolegend	Cat #115519; RRID:AB_313654
Anti-mouse CD335 (PE/Cyanine7)	Biolegend	Cat #137617; RRID:AB_11218594
7AAD viability staining solution	Biolegend	Cat #420403
Bacterial and virus strains		
Adeno-CMV-CRE	Iowa Viral Vector Core	VVC-U of Iowa-5
Biological samples		
Human lung adenocarcinoma samples	NRS Lothian Bioresource	Bioresource ref: SR1382
Human lung squamous carcinoma-in-situ samples	UCLH early lung cancer surveillance program	REC ref: 06/Q0505/12 and 01/0148
Chemicals, peptides, and recombinant proteins		
Anti-Digoxigenin-AP	Roche	Cat #11093274910
Proteinase K	Roche	Cat #03115879001
NBT	Promega	Cat #S3771
BCIP	Roche	Cat #16853423
Experimental models: Organisms/strains		
B6.129-*Tlr2^tm1Kir^*/J	Jax.org	004650
B6.129S4-*Kras^tm4Tyj^*/J	Jax.org	008179
B6.129- *Kras*^*tm4Tyj*^ *Trp53*^*tm1Brn*^/J	Jax.org	032435
Oligonucleotides		
Primers for qPCR; see Table S1	This paper	N/A
gRNA sequence for pSECC; see Table S2	This paper	N/A
Primers for mouse Tlr2 RNA-ISH; see Table S3	This paper	N/A
Software and algorithms		
QuPath	Bankhead et al., 2017	https://qupath.github.io

## Highlights

- TLR2 is associated with improved survival and premalignant regression in NSCLC
- TLR2 mediates oncogene-induced senescence and the SASP in early lung tumors
- The Tlr2-mediated SASP recruits myeloid cells and promotes senescence surveillance
- Targeting Tlr2 therapeutically reduces lung tumor growth in mouse models
