# Supplementary material for: Toll-like receptor 2 orchestrates a tumor suppressor response in non-small cell lung cancer
Source: Cell Rep. Author manuscript; Available in PMC 2023 May 19. (PMC10197427; doi:10.1016/j.celrep.2022.111596)
Supplement: supplemental data [file NIHMS1888916-supplement-supplemental_data.zip › 1-s2.0-S2211124722014619-mmc1.pdf]

Cell Reports, Volume *41*

Supplemental information

Toll-like receptor 2 orchestrates a tumor suppressor response in non-small cell lung cancer

Fraser R. Millar, Adam Pennycuick, Morwenna Muir, Andrea Quintanilla, Priya Hari, Elisabeth Freyer, Philippe Gautier, Alison Meynert, Graeme Grimes, Carla Salomo Coll, Sofia Zdral, Stella Victorelli, Vitor H. Teixeira, John Connelly, João F. Passos, Marian A. Ros, William A.H. Wallace, Margaret C. Frame, Andrew H. Sims, Luke Boulter, Sam M. Janes, Simon Wilkinson, and Juan Carlos Acosta

Supplementary figures

Supplementary figure 1

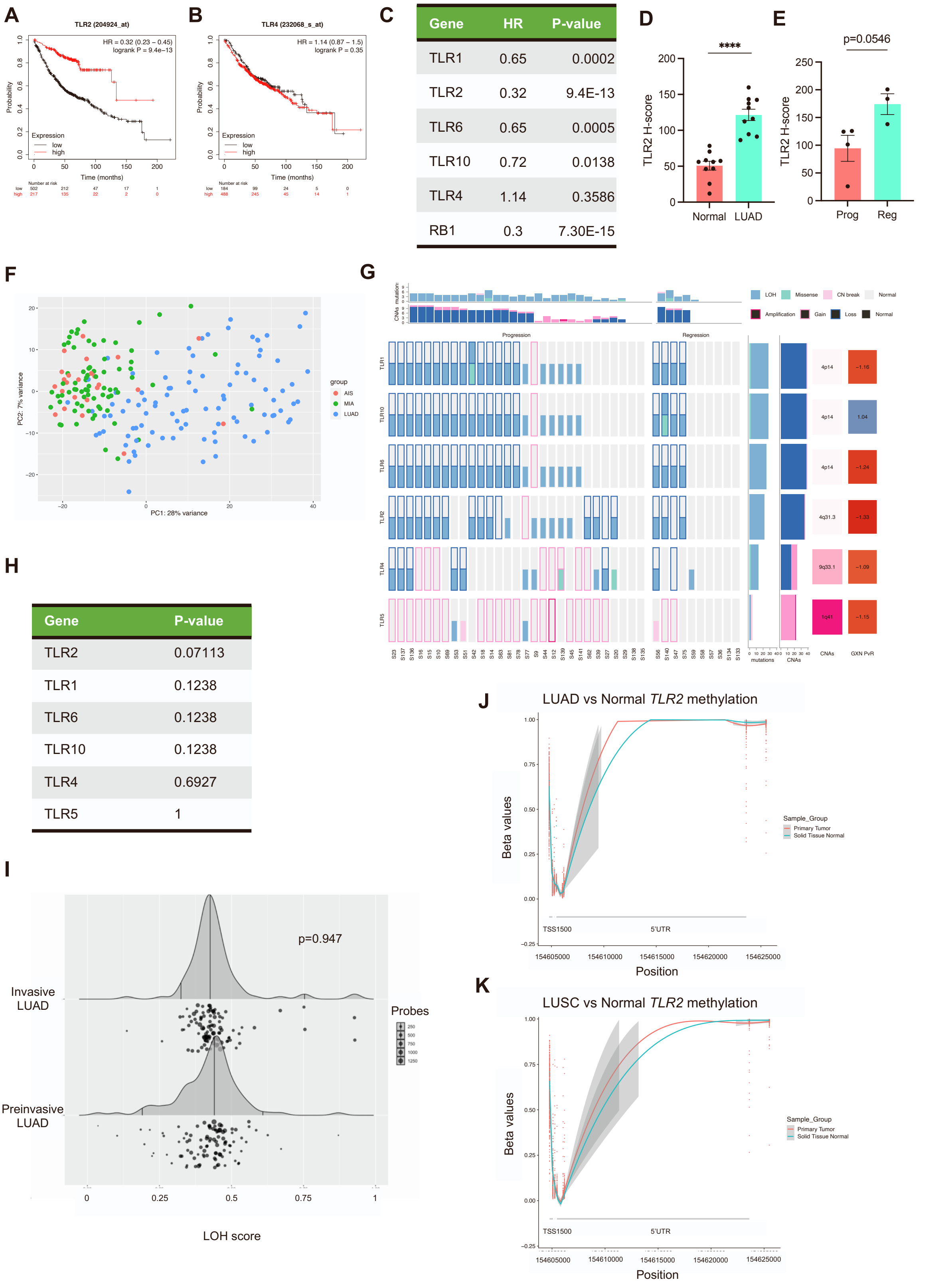

Gene	HR	P-value
TLR1	0.65	0.0002
TLR2	0.32	9.4E-13
TLR6	0.65	0.0005
TLR10	0.72	0.0138
TLR4	1.14	0.3586
RB1	0.3	7.30E-15

Gene	P-value
TLR2	0.07113
TLR1	0.1238
TLR6	0.1238
TLR10	0.1238
TLR4	0.6927
TLR5	1

Supplementary figure 1: TLR2 genetic, transcriptomic and epigenetic alterations in non-small cell lung cancer. Related to figure 1. A, Kaplan-Meier survival plot showing the survival differences between high and low TLR2 expression and **B**, TLR4 expression in lung adenocarcinoma samples from KMplot.com (corresponding to 866 patients from the GEO, EGA and TCGA). **C**, Table showing hazard ratios (HR) and corresponding p-value of each indicated gene (including TLR2 and its dimerization partners TLR1, 6 and 10, and the non-TLR2 associated plasma membrane Toll-like receptor TLR4) in human lung adenocarcinoma. TLR2 shows the lowest HR, which compares with the well characterized tumor suppressor gene RB1. The effect in lung adenocarcinoma is specific to the TLR2 network as the other plasma membrane TLR (TLR4) does not show prognostic value. **D**, IHC quantification of TLR2 expression in LUAD lesions and paired normal tissue. Statistical analysis was performed using the paired Student's *t*-test. **** $p<0.0001$. **E**, IHC quantification of TLR2 expression in preinvasive LUSC lesions that either progressed to cancer (Prog) or regressed to normal epithelium (Reg). Statistical analysis was performed using the Student's *t*-test. **F**, Principal component analysis (PCA) plot of gene expression from preinvasive LUAD lesions (AIS (adenocarcinoma in situ), MIA (minimally invasive adenocarcinoma)) and invasive lung adenocarcinoma (LUAD) revealing clustering of preinvasive LUAD lesions (AIS and MIA). **G**, Genomic aberrations affecting TLR genes in preinvasive LUSC lesions. The mutational status is shown for 6 key plasma membrane TLR genes including those associated with the TLR2 signalling network (TLR2, 1, 6 and 10) and non-TLR2 associated plasma membrane TLRs (TLR4 and 5). Mutations and CNAs are shown for each of 29 progressive and 10 regressive samples. Loss of heterozygosity (LOH) events are shown as mutations to avoid confusion with copy-number loss, relative to ploidy. The GXN PvR column displays the fold change in expression of each gene between progressive and regressive samples, defined in a partially overlapping set of 18 samples. Significant genes, defined as $FDR < 0.05$, are highlighted in blue. **H**, Table with p-values calculated using Fisher's exact test to assess the likelihood of LOH being associated with preinvasive LUSC lesion progression. **I**, *TLR2* locus LOH frequency analyzed in whole exome sequencing from preinvasive and invasive LUAD samples. The mean LOH score over the *TLR2* locus was determined and statistical analysis was performed using the two-sided Wilcox rank sum test. Stat=4780, p-0.947. Methylation patterns are shown for *TLR2* in **J**, LUAD and **K**, LUSC and compared to adjacent normal tissue from TCGA data. TSS1500 represents the Transcription Start Site regions. The x-axis shows the genomic location of each probe, and the y-axis shows the probe values for each sample, colored as tumor (orange) and normal tissue (green). Loess lines for each sample are shown with error bars in grey.

33

Supplementary figure 2

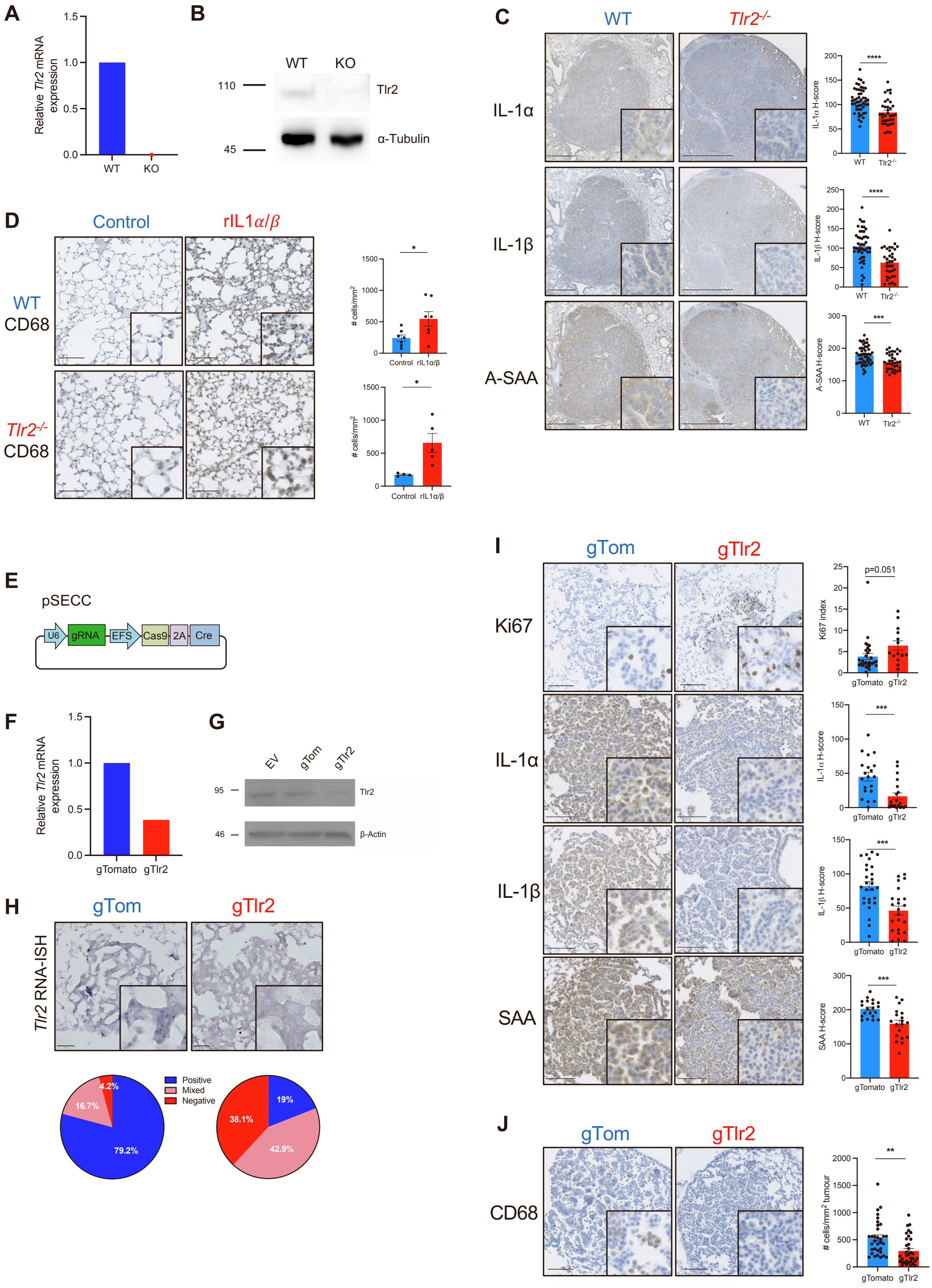

34

35

Supplementary figure 2: Mouse model validation and SASP expression analysis in *Trp53* null tumors and gTlr2 pSECC tumors. Related to figure 2 and 3. A, Relative *Tlr2* mRNA expression in RNA samples extracted from *Tlr2* wild-type (WT) and knock-out (KO) murine lung tissue. **B**, Western blot analysis for Tlr2 and alpha tubulin (loading control) in protein extracted from *Tlr2* wild-type (WT) and knock-out (KO) murine lung tissue. **C**, Representative IHC staining of lung tumors from $Kras^{LSL\text{-}G12D/+}$;$Trp53^{fl/fl}$ mice on either a wild-type (WT) or *Tlr2* null ($Tlr2^{-/-}$) background for the SASP factors interleukin-1-alpha (IL-1α), interleukin-1-beta (IL-1β) and serum amyloid A (SAA), with corresponding quantification. n=7-10 mice per group (five tumors per mouse analyzed). Statistical analysis was performed using the Student's *t*-test. ***p<0.001, ****p<0.0001. Scale bars 500um. **D**, Representative IHC staining for CD68 in lung tissue from $Tlr2^{+/+}$ (WT) and $Tlr2^{-/-}$ mice intranasally inoculated with either PBS (control) or recombinant interleukin-1-alpha (rIL-1α) and recombinant interleukin-1-beta (rIL-1β), with corresponding quantification. Statistical analysis was performed using the Student's *t*-test. *p<0.05. Scale bars 100um. **E**, *Tlr2* targeting gRNA were cloned into pSECC lentiviral plasmids to allow concurrent *Tlr2* deletion and $Kras^{G12D}$ activation in lung epithelial cells only following intranasal inoculation of $Kras^{LSL\text{-}G12D/+}$ mice. **F**, qRT-PCR and **G**, Western blot analysis of *Tlr2* mRNA and protein extracted from mouse embryonic fibroblasts (MEFs) infected with either non-target pSECC lentivirus (gTom) or Tlr2 targeting pSECC lentivirus (gTlr2). Of note there is no selection cassette in the pSECC plasmid hence residual *Tlr2* expression from non-infected cells. **H**, Representative images of *Tlr2* RNA in situ hybridization (RNA-ISH) performed on lung tumors targeted with control (gTom) or Tlr2 targeting pSECC lentivirus (gTlr2). Scale bars 50um. Tumors were scored as either positive (100% expression), mixed (>30% expression) and negative (<30% expression). **I**, Representative IHC staining and quantification for Ki67 and the SASP factors interleukin-1-alpha (IL-1α), interleukin-1-beta (IL-1β), acute-phase serum amyloid A (A-SAA) on lung tumors from $\mathrm{Kras}^{LSL\text{-}G12D/+}$ mice that either received a non-target pSECC lentivirus (gTom) or *Tlr2* targeting pSECC lentivirus (gTlr2) with corresponding quantification. Statistical analysis was performed using the Student's *t*-test. ***p<0.001. Scale bars 100um. **J**, Representative IHC staining and quantification for the CD68 in lung tumors from $Kras^{LSL\text{-}G12D/+}$ mice inoculated with either gTomato (gTom) or gTlr2 expressing lentivirus. n=8 mice per group. Statistical analysis was performed using the Student's *t*-test. ***p<0.001, ****p<0.0001. Scale bars 100um.

Supplementary figure 3

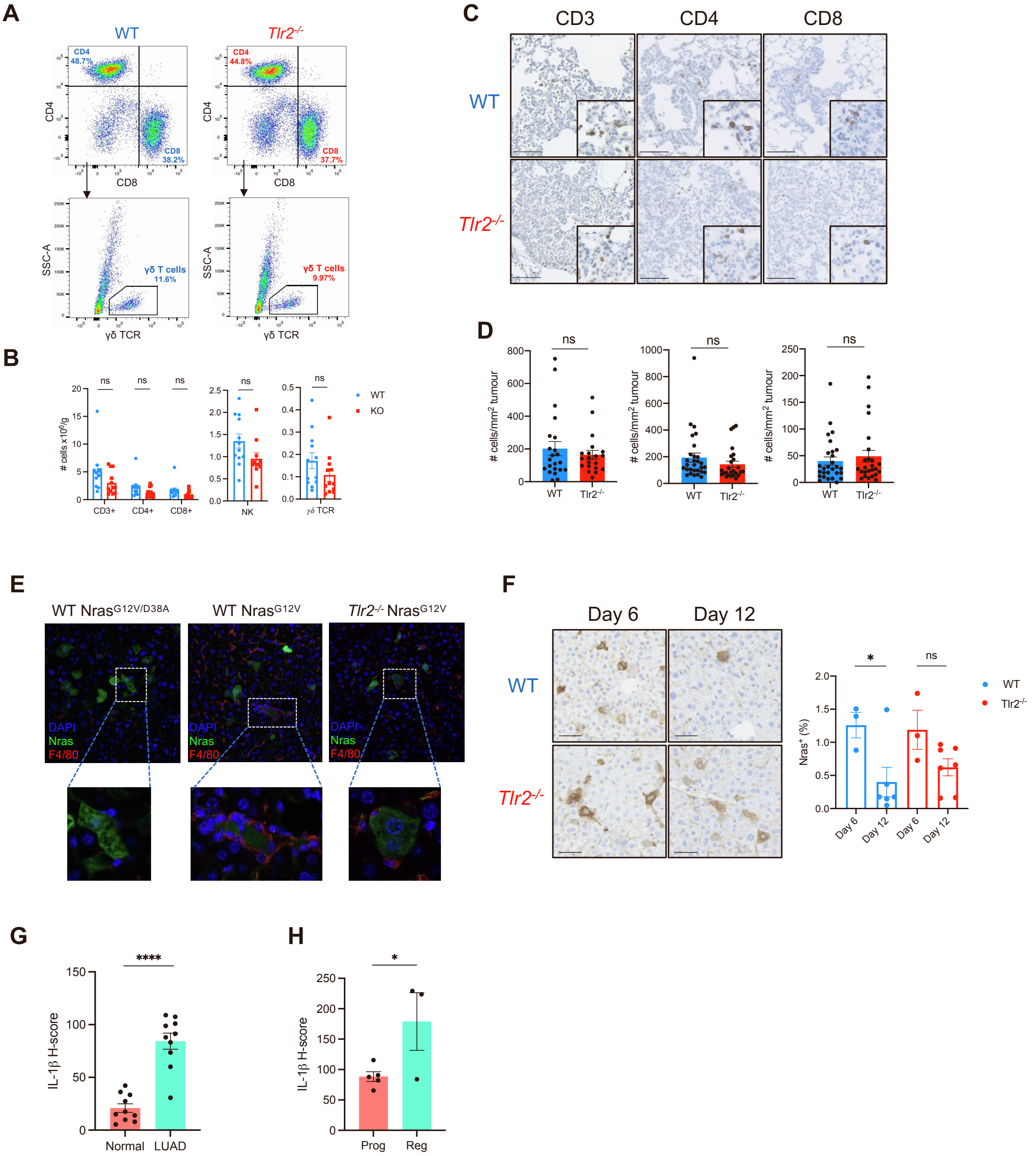

Supplementary figure 3: Immune cell analysis in lung tumors and senescence surveillance analysis in hydrodynamic tail vein injection model. Related to figure 3. A, Representative flow cytometry analysis plots of lymphoid populations from whole lung single cell suspensions from lung tumor bearing WT or *Tlr2*$^{-/-}$ mice. Percentage denotes percentage of parent population. **B**, Corresponding quantification of lymphoid cells from WT (blue) and *Tlr2*$^{-/-}$ (red) mice. CD3+ - total T-cells, CD4+ - CD4 T-cells, CD8+ - CD8 T-cells, NK – natural killer cells, $\gamma\delta$ TCR - $\gamma\delta$ T-cells. n=12 mice per group. Statistical analysis was performed using the Student's *t*-test. ns – non-significant. **C**, Representative IHC staining for the pan T-cell marker CD3 and specific T-cell markers CD4 and CD8 in WT and *Tlr2*$^{-/-}$ tumors with corresponding quantification in **D**. n=5-6 mice per group (five tumors per mouse analyzed). Statistical analysis was performed using the Student's *t*-test. ns – non-significant. Scale bars 100um. **E**, Representative co-immunofluorescence staining for DAPI (blue), Nras (green) and F4/80 (red) on liver section from WT or *Tlr2*$^{-/-}$ mice six days after hydrodynamic delivery of oncogenic Nras (*Nras*^{G12V}) or negative control (*Nras*$^{G12V/D38A}$) expressing transposons. **F**, Liver IHC staining for Nras from WT or *Tlr2*$^{-/-}$ mice six and twelve days after hydrodynamic delivery of *Nras*^{G12V} expressing transposons, with corresponding quantification. Statistical analysis was performed using the Student's *t*-test. ns – non-significant, *p<0.05. Scale bars 50um. **G**, Quantification of IL1B IHC in paired normal epithelium (Normal) and lung adenocarcinoma (LUAD). Statistical analysis was performed using the paired Student's *t*-test. ****p<0.0001. **H**, Quantification of IL1B IHC in preinvasive LUSC lesions that either progressed to cancer (Prog) or regressed to normal epithelium (Reg). Statistical analysis was performed using the Student's *t*-test. *p<0.05.

Supplementary figure 4

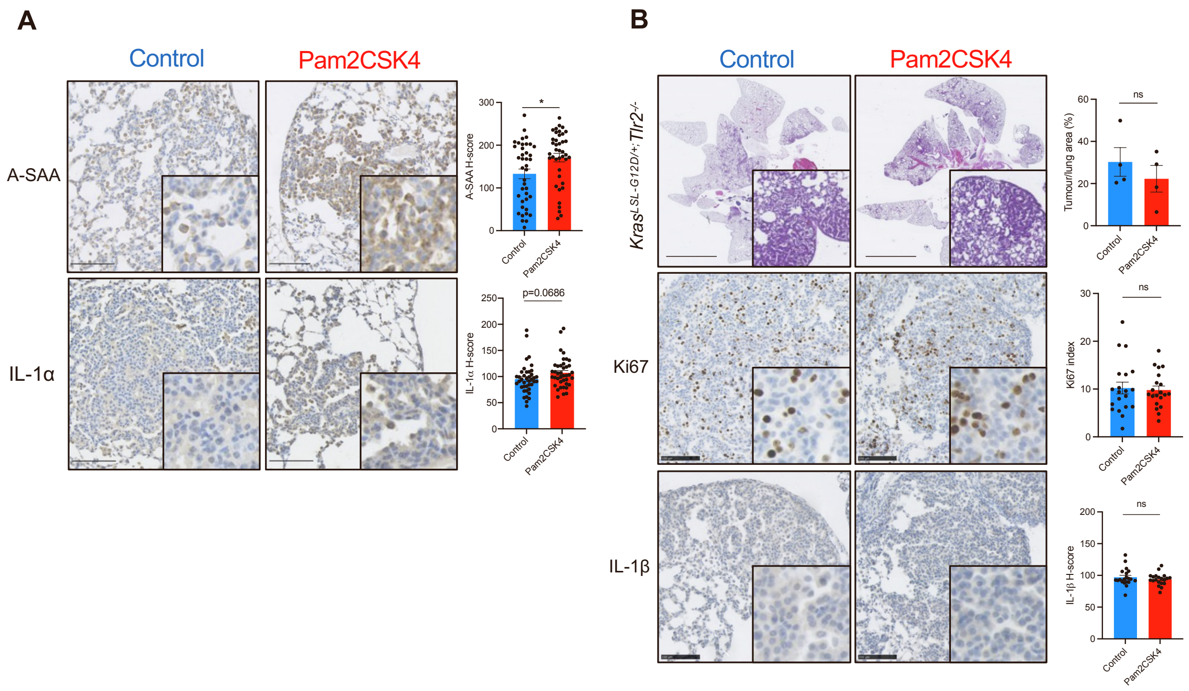

Supplementary figure 4: SASP expression analysis after Pam2CSK4 treatment and Pam2CSK4 treatment in *Tlr2* null mice. Related to figure 4. A, Representative IHC staining for acute phase serum amyloid A (A-SAA) and interleukin 1 alpha (IL-1α), with corresponding quantification. n=8 mice per group (five tumors per mouse analyzed). Statistical analysis was performed using the Student's *t*-test. *p<0.05. Scale bars 100um. **B**, Representative H&E and IHC staining for Ki67 and interleukin-1-beta (IL-1*β*) in lung tumors from *Tlr2* null $Kras^{LSL\text{-}G12D/+}$ mice ($Kras^{LSL\text{-}G12D/+}$;$Tlr2^{-/-}$) after either control or Pam2CSK4 treatment, with corresponding quantification. n=4 mice per group (five tumors per mouse analyzed). Statistical analysis was performed using the Student's *t*-test. ns – non-significant. Scale bars 5mm for H&E images, 100um for IHC images.

SUPPLEMENTARY TABLES

Table 1 – qRT-PCR primers, Related to STAR Methods

PRIMER	SEQUENCE
**Mouse beta actin F/W**	GGCACCACACCTTCTACAA
**Mouse beta actin R/V**	GTGGTGGTGAAGCTGTAGCG
**Mouse Tlr2 F/W**	TCCTGCGAACTCCTATCCTTTACTA
**Mouse Tlr2 R/V**	TGGCCAGTCAACCAGGATTTG

Table 2 - gRNA sequences for pSECC constructs, Related to STAR Methods

gRNA ID	TARGET SEQUENCE
**gTomato**	GGCCACGAGTTCGAGATCGAGGG
**gTlr2**	CCTGGAGGTTCGCACACGCT

Table 3 – Primers for mouse Tlr2 DNA amplification (RNA-ISH) , Related to STAR Methods

PRIMER	SEQUENCE
**Forward**	AGCCTGAAGTGGGAGAAGTC
**Reverse**	ATTTAGGTGACACTATAGAACTACAGTGAGCAGGATTCCC
